# Pilot Study of an Individualised Early Postpartum Intervention to Increase Physical Activity in Women with Previous Gestational Diabetes

**DOI:** 10.1155/2012/892019

**Published:** 2012-04-04

**Authors:** Harold David McIntyre, Ann Peacock, Yvette D. Miller, Denise Koh, Alison L. Marshall

**Affiliations:** ^1^The University of Queensland, St. Lucia, QLD 4072, Australia; ^2^Mater Medical Research Institute, Raymond Terrace, South Brisbane, QLD 4101, Australia; ^3^The National University of Malaysia, Bangi, 43600 Selangor, Malaysia; ^4^Queensland University of Technology, Brisbane, QLD 4001, Australia

## Abstract

Optimal strategies to prevent progression towards overt diabetes in women with recent gestational diabetes remain ill defined. We report a pilot study of a convenient, home based exercise program with telephone support, suited to the early post-partum period. Twenty eight women with recent gestational diabetes were enrolled at six weeks post-partum into a 12 week randomised controlled trial of Usual Care (*n* = 13) versus Supported Care (individualised exercise program with regular telephone support; *n* = 15). Baseline characteristics (Mean ± SD) were: Age  33 ± 4  years; Weight 80 ± 20 kg and Body Mass Index (BMI) 30.0 ± 9.7 kg/m^2^. The primary outcome, planned physical activity {Median (Range)}, increased by 60 (0–540) mins/week in the SC group versus 0 (0–580) mins/week in the UC group (*P* = 0.234). Walking was the predominant physical activity. Body weight, BMI, waist circumference, % body fat, fasting glucose and insulin did not change significantly over time in either group. This intervention designed to increase physical activity in post-partum women with previous gestational diabetes proved feasible. However, no measurable improvement in metabolic or biometric parameters was observed over a three month period.

## 1. Background

 Strategies to prevent the progression from impaired glucose tolerance to overt (principally type 2) diabetes in middle-aged and older adults have been developed by a number of groups worldwide, drawing on the results of major randomised controlled trials [1–3]. Women with previous gestational diabetes (GDM) are known to be at high risk of progression to type 2 diabetes [4]. However, strategies for diabetes prevention for women with previous GDM in the period immediately following pregnancy are less well defined. The TRIPOD [5] and PIPOD studies [6] demonstrated that thiazolidinedione (TZD) therapy could delay progression to diabetes in a high risk group of women. Some benefits have also been suggested for metformin by Ratner and colleagues [7] in women with previous GDM (mean age at study entry 43 years) who participated in the Diabetes Prevention Program (DPP). In women with previous GDM, metformin led to a 50% reduction of the risk of progression from impaired glucose tolerance to overt diabetes, whereas lifestyle intervention was associated with a 53% risk reduction. However, medication-based strategies may not be appropriate for women of child-bearing age and are unlikely to be feasible or desirable on a broader scale.

Anecdotally, the pressures of caring for a new baby tend to dominate the early postpartum period, with Australian women potentially experiencing difficulty focusing on their own long-term health, and specifically their exercise, in this context. This belief is supported by a recent qualitative study conducted in the USA that found that having young children/child was a major barrier to an active lifestyle in the first 12 months postpartum [8]. Our recent work [9] has demonstrated that women with previous GDM frequently have ongoing deficits in health promoting physical activity. By contrast, the recent findings of Retnakaran et al. [10] were more positive, suggesting some improvement in physical activity following a GDM pregnancy.

Changes in lifestyle patterns at this time might potentially prove to be valuable in preventing longer term progression towards diabetes, as well as influencing the woman's entire family towards adopting health promoting behaviours. However, Cheung et al. have reported little success with a group intervention that used patient-centred counselling [11] or more recently with a pedometer-linked programme [12]. In contrast, several intervention studies based on the Social Cognitive Theory [13] have demonstrated success in increasing and even maintaining physical activity among individuals with type 2 diabetes [14]. This pilot study sought to evaluate the feasibility and efficacy of an individualised programme, based on the social cognitive theory, to assist women to be more physically active in the early post natal period.

## 2. Research Design and Methods

 The protocol was approved by Hospital and University Human Research Ethics Committees. Participants consented in writing after appropriate verbal and written explanation of the study. The study was registered with the Australian and New Zealand Clinical Trials Registry: ACTRN 12608000280303.

Seventy-two women were approached to join the trial prior to six weeks postpartum. Forty-three women refused participation and one was excluded due to detection of overt diabetes on the entry oral glucose tolerance test (OGTT), leaving 28 randomised participants. At six weeks after delivery of the index pregnancy complicated by GDM, participants underwent baseline assessment. Parameters assessed included a 75 g OGTT, fasting insulin, body weight and height using standardised instruments, and body composition using bioimpedance methodology. Insulin resistance was estimated using the HOMA-IR equation [15] (HOMA-IR = Fasting insulin (*μ*U/mL) × Fasting glucose (mmol/L)/22.5). Physical Activity was assessed using the validated Australian Women's Activity Survey [16].

Women were then randomly assigned to one of two groups. The Usual Care group (“UC”, *n* = 13) received brief printed materials outlining the importance of diet and exercise for the prevention of future diabetes. The Supported Care (“SC”, *n* = 15) group underwent an initial face-to-face consultation with an exercise physiologist where specific, individualised goals for initiating and maintaining regular health-enhancing physical activity were developed. Consistent with current physical activity guidelines a physical activity target of 150 mins/wk was set, to be achieved gradually over the 12 weeks intervention through activities acceptable to the individual. The exercise physiologist contacted each woman in the SC group weekly by telephone for the next four weeks and then every 2 weeks thereafter to assess progress, promote accountability, and to provide tailored expert support for recognising and overcoming experienced constraints to physical activity behaviour change.

Twelve weeks following baseline assessment (total 18 weeks postpartum), both groups underwent repeat examinations as noted above, except that samples for fasting glucose and insulin alone were taken without a repeat OGTT. The primary outcome measure was change in self-reported physical activity. Secondary outcomes were change in insulin resistance (HOMA—IR), change in weight, and changes in body composition.

Statistical analyses were performed using data from those women who completed both assessments *n* = 11 “UC” and *n* = 14 “SC” women. All comparisons between the UC and SC groups consider differences between these groups in the change or “Delta” (Delta = Value_18  weeks  post  partum_ − Value_6  weeks  post  partum_) in each variable between six and 18 weeks postpartum. Statistical comparisons have been performed using unpaired *t*-tests for normally distributed variables and Mann Whitney *U* tests for nonnormally distributed variables. Categorical variables were analysed using Fisher's exact test due to small cell sizes. Significance was accepted at the 5% level for two-tailed analysis for all variables.

## 3. Results

Typical of an Australian GDM cohort, the women were generally in their early thirties and their mean body mass index (BMI) at six weeks postpartum was in the obese range. Importantly there were no significant differences between the study groups demographic, physical activity or insulin resistance at baseline (see Table 1). Two UC and one SC participant dropped out of the study prior to the follow-up assessment.

Consistent with previous studies, the physical activity data were nonnormally distributed (see Table 2). Median (range) planned physical activity increased by 60 (0–540) mins/wk in the SC group versus 0 (0–580)  mins/wk in the UC group, but this change was not statistically significant (*P* = 0.234, Mann-Whitney *U* test). The change observed in the SC group's physical activity comprised mostly increased planned walking. A pre defined categorical analysis examined differences between SC and UC groups in the proportion of women increasing planned physical activity by >60 mins/wk; 67% of women who received SC achieved this criterion compared to 31% of women who received UC. Despite this, most women regardless of group allocation failed to reach the recommended physical activity level of 150 mins/wk (see Table 2).

Metabolic assessments revealed no changes in weight or insulin resistance in either group (see Table 2). Body composition (% lean mass, % fat mass) was also unchanged. Breast feeding status (full/partial/nil) was also noted at six and 18 weeks postpartum. Weight loss and other metabolic parameters did not differ between breastfeeding groups.

Open-ended feedback regarding the intervention programme was obtained from the SC group. Whilst most women responded positively to the programme, many commented that the starting point of six weeks postpartum was too early for maximum benefit, as they were still adapting to life with a new baby and found it difficult to focus on personal lifestyle changes such as increasing physical activity at that time.

## 4. Discussion

This pilot study was designed to evaluate and refine a potential early postpartum intervention designed to increase physical activity in women with previous gestational diabetes for future dissemination and evaluation. Our findings suggest that a postpartum programme designed to encourage and assist women with prior GDM to be more physically active is feasible.

Specific strengths of our study included the randomized design and good overall retention of participants. Weaknesses included relatively poor recruitment rates, anecdotally contributed to by the predominance of “baby-related” concerns in early postpartum period, short duration of followup, and small total study cohort.

As noted in Tables 1 and 2, there was great variability in physical activity both at baseline and at followup, with many women reporting essentially zero planned physical activity. The variance in all biophysical study measures was large, in particular for HOMA-IR where the standard deviations approached or exceeded the mean values. In designing future studies, it may be worthwhile to stratify women according to BMI at entry, as this is likely to be a major factor influencing the degree of insulin resistance.

Notwithstanding the timing of commencement, the intervention was well received. Anecdotally, women were happy that their potential health problems were being addressed in an organised programme. Although changes in physical activity between groups did not reach statistical significance, the proportion of women increasing their physical activity by >60 mins/wk in the SC group was twice that of women in the UC group. If confirmed in a larger study sample and maintained over a longer period of time, this would provide significant health benefits [17].

Commencement of programmes designed to increase physical activity in the early postpartum period has some potential advantages in terms of capitalizing on the increased motivation often seen in pregnancy. However, the focus of attention frequently shifts to the baby at this stage, making alterations in ingrained maternal behaviours potentially difficult to achieve. The emotional stress of adapting to a new baby and the fear of receiving a diagnosis of diabetes are key barriers to follow-up care for GDM [18].

As noted previously, other studies of interventions in the postpartum period [10–12] have met with limited and variable success and the optimal timing and content of postpartum programmes remains undefined. Group-based programmes may help increase motivation [11, 12] but achieving “buy in” and maintaining participation appears challenging. Despite the theoretical advantage of commencing diabetes prevention at an earlier stage of pathophysiology, practical barriers may make women more resistant to change at this stage of the life cycle.

Despite some evidence of increased physical activity, measures of glucose metabolism were not altered by this intervention over a three-month period. This was not unexpected given the small sample size and short duration of the study, but we noted absolutely no trends in favour of metabolic improvement. Weight and body composition were also unchanged. Although early postpartum breast feeding status did not appear to influence our findings, the potential importance of breast feeding in longer term diabetes prevention has also been noted in a recent review [18].

Alternatively, one could argue in favour of pharmacologic prevention of progression towards diabetes following GDM, citing the results of the TRIPOD [5], PIPOD [6], and DPP [7] studies. However, thiazolidinediones are rapidly disappearing from the pharmacotherapy of type 2 diabetes due to an unfavourable risk/benefit profile and their potential use in diabetes prevention appears severely limited. Metformin was reported to be equally efficacious as an intensive diet/lifestyle programme in women with previous GDM who participated in the DPP [7], but this finding relates to much older women (mean age 46 years at study entry), rather than those in the early postpartum period. For large scale intervention, lifestyle measures appear intrinsically more attractive, though metformin may still deserve consideration in those struggling to make effective lifestyle changes.

Further research is warranted to improve the physical activity levels and general health of women with previous GDM. We suggest that studies combining physical activity and dietary interventions may potentially offer greater benefits and we are currently planning such studies, using the pilot data reported in this paper. We hope that our findings will also assist other researchers in determining in the design and conduct of more definitive studies, in particular by allowing *pre hoc* power calculations to be performed in a more robust fashion.

## Figures and Tables

**Table 1:** Prestudy characteristics of women, at the baseline visit (six weeks postpartum), divided by treatment group.

Parameter	Usual Care group (*n* = 13)	Supported Care group (*n* = 15)
	Mean ± SD	Mean ± SD
Age—years	31.5 ± 3.9	34.6 ± 4.4
Weight—kg	80.3 ± 17.4	79.3 ± 20.7
BMI—kg/m^2^	30.3 ± 7.4	30.6 ± 8.5
Waist circumference (cm)	96.0 ± 11.0	97.6 ± 15.2
% Body fat	32.7 ± 8.1	33.5 ± 8.3
Fasting glucose—mmol/L;	4.7 ± 0.7	4.6 ± 0.7
Fasting insulin—*μ*U/mL;	8.4 ± 7.5	8.4 ± 7.5
HOMA—IR	1.9 ± 2.0	1.8 ± 1.8

	*n* (%)	*n* (%)
Parity > 1	9 (69%)	9 (60%)
Education > high school	8 (62%)	9 (60%)

Planned physical activity (mins/wk)	Median (range)	Median (range)
	0 (0–420)	0 (0–300)

**Table 2:** Changes in physical activity, weight, and insulin resistance of women between baseline (six weeks postpartum) and followup (18 weeks postpartum) by treatment group. All changes calculated as (Value_18  weeks  post  partum_ − Value_6  weeks  post  partum_).

Parameter	Usual Care group	Supported Care group
	(*n* = 11 at end of study)	(*n* = 14 at end of study)
	Median (range)	Median (range)
Change in planned physical activity (mins/wk)	0 (0–580)	60 (0–540)
Change in planned walking (mins/wk)	0 (0–360)	60(0–345)

	%	%
Change in planned physical activity > 60 mins/wk	31%	67%
Meets physical activity goal of 150 mins/wk	Never: 54%	Never 53%
	18 weeks only 31%	18 weeks only 40%
	6 & 18 weeks 15%	6 & 18 weeks 7%

	Mean ± SD	Mean ± SD
Change in Weight (kg)	0.22 ± 4.2	0.97 ± 3.7
Change in Waist circumference (cm)	−3.6 ± 7.3	−0.35 ± 3.8
Change in % Body Fat	−1.2 ± 3.3	1.0 ± 4.4
Change in fasting glucose (mmol/L)	0.12 ± 0.42	0.25 ± 0.56
Change in fasting insulin (*μ*U/mL)	0.06 ± 3.89	1.49 ± 4.23
Change in HOMA IR	−0.08 ± 1.02	0.43 ± 1.28

## Abbreviations

BMI: Body mass index
GDM: Gestational diabetes mellitus
HOMA: Homeostasis model assessment
IR: Insulin resistance
OGTT: Oral glucose tolerance test
SC: Supported care
UC: Usual care.
